# An Orphan VrgG Auxiliary Module Related to the Type VI Secretion Systems from *Pseudomonas ogarae* F113 Mediates Bacterial Killing

**DOI:** 10.3390/genes14111979

**Published:** 2023-10-24

**Authors:** David Durán, David Vazquez-Arias, Esther Blanco-Romero, Daniel Garrido-Sanz, Miguel Redondo-Nieto, Rafael Rivilla, Marta Martín

**Affiliations:** 1 Departamento de Biología, Facultad de Ciencias, Universidad Autónoma de Madrid, Darwin, 2, 28049 Madrid, Spain; david.duran@uam.es (D.D.); david.vazquez@uam.es (D.V.-A.); esther.blanco-romero@aphea.bio (E.B.-R.); daniel.garridosanz@unil.ch (D.G.-S.); miguel.redondo@uam.es (M.R.-N.); rafael.rivilla@uam.es (R.R.); 2 Department of Fundamental Microbiology, University of Lausanne, 1015 Lausanne, Switzerland

**Keywords:** T6SS, *Pseudomonas ogarae* F113, VgrG auxiliary module

## Abstract

The model rhizobacterium *Pseudomonas ogarae* F113, a relevant plant growth-promoting bacterium, encodes three different Type VI secretion systems (T6SS) in its genome. In silico analysis of its genome revealed the presence of a genetic auxiliary module containing a gene encoding an orphan VgrG protein (VgrG5a) that is not genetically linked to any T6SS structural cluster, but is associated with genes encoding putative T6SS-related proteins: a possible adaptor Tap protein, followed by a putative effector, Tfe8, and its putative cognate immunity protein, Tfi8. The bioinformatic analysis of the VgrG5a auxiliary module has revealed that this cluster is only present in several subgroups of the *P*. *fluorescens* complex of species. An analysis of the mutants affecting the *vgrG5a* and *tfe8* genes has shown that the module is involved in bacterial killing. To test whether Tfe8/Tfi8 constitute an effector–immunity pair, the genes encoding Tfe8 and Tfi8 were cloned and expressed in *E*. *coli*, showing that the ectopic expression of *tfe8* affected growth. The growth defect was suppressed by *tfi8* ectopic expression. These results indicate that Tfe8 is a bacterial killing effector, while Tfi8 is its cognate immunity protein. The Tfe8 protein sequence presents homology to the proteins of the MATE family involved in drug extrusion. The Tfe8 effector is a membrane protein with 10 to 12 transmembrane domains that could destabilize the membranes of target cells by the formation of pores, revealing the importance of these effectors for bacterial interaction. Tfe8 represents a novel type of a T6SS effector present in pseudomonads.

## 1. Introduction

The Type VI Secretion Systems (T6SSs) are nanomachine systems that translocate specific proteins directly into target cells [1]. These systems were originally described in *Vibrio cholerae* [2] and *Pseudomonas aeruginosa* [3]. T6SSs are present in more than 25% of gram-negative bacteria, mostly confined to the phylum Proteobacteria [4,5] and frequently encoding more than one T6SS in their genome [6,7]. Although it has been characterized as a classical virulence factor against eukaryotic cells [1,8,9] and plants [10,11,12], the relevance of T6SSs resides mainly with its anti-prokaryotic activity [13,14,15,16].

The common genetic organization of the T6SSs groups the structural proteins of the system with well-defined functions in genomic clusters that generally comprise 13 to 15 genes [7,17,18,19]. The genes encoding the T6SS effectors and their cognate immunity proteins are commonly linked to the *hcp* and/or *vgrG* genes within T6SS clusters [20,21,22] or in the islands/auxiliary modules harboring orphan *vgrG* or *hcp* genes [23,24]. Most T6SS effectors are antibacterial toxins that have been described in bacterial pathogens such as *V. cholerae* and *P. aeruginosa* [25,26,27]; nevertheless, T6SS antifungal effectors have been found in *Serratia marcescens* as growth inhibitors of pathogenic species [16]. Genes encoding effectors are frequently found adjacent to genes encoding immunity proteins, usually forming transcriptional units [13,28,29,30,31]. Genes encoding orphan VgrG proteins, not genetically linked to any T6SS structural cluster, have also been described for several T6SS-containing bacteria [24,32,33]. These orphan VgrG-encoding genes are frequently found dispersed in the genome, not being directly associated with defined T6SSs clusters. Interestingly, some of them conform to a VgrG island, auxiliary module, orphan effector island, or effector island [24,33,34,35,36] where the *vgrG* genes are ligated to genes encoding adaptor proteins (commonly Tap proteins) and effector–immunity pairs.

In the *Pseudomonas* genus, the T6SS from *P. aeruginosa* is a well-studied system that plays an essential role in several biological processes [13,36,37,38,39,40,41] and, to a lesser extent, in the case of *P. putida* as a system with activity against phytopathogens [22]. In other species from this genus, the T6SS has been implicated in siderophore production [42], bacteria colony invasion [43], and rhizosphere colonization [44].

Additionally, T6SSs have been described in species belonging to the *P. fluorescens* complex of species [45], playing a role in the rhizosphere and insect gut environment [46,47] or releasing anti-bacterial toxins [48,49]. We have recently described a role for the T6SS of *Pseudomonas ogarae* F113 in the colonization of the rhizosphere [44,50]. These findings highlight the importance of T6SSs in environmental adaption. In addition to the structural elements of T6SSs, *P*. *ogarae* F113 possesses five orphan *vgrG* genes across its genome [51]. In this work, we analyze one such auxiliary module which contains a *vgrG* gene associated with a gene encoding an adaptor Tap protein and genes encoding a putative effector–immunity pair named Tfe8-Tfi8 [44]. Our results show that this auxiliary module is functional in bacterial killing, defining a novel type of a Type 6 effector protein.

## 2. Materials and Methods

### 2.1. Bacterial Strains and Culture Conditions

The bacterial strains, vectors, plasmids, and derivative constructions employed in this study are listed in Supplementary Table S1, which includes relevant information associated with each strain. *Pseudomonas ogarae* strains were grown in Sucrose–Asparagine medium (SA) [52] at 28 °C. The *Escherichia coli* strains were grown in Lysogeny Broth (LB) medium [53] at 37 °C. *E*. *coli* was used for cloning purposes (DH5α cells) and protein expression [BL21(DE3) cells]. Antibiotics, inducers, and repressors were added as required at the following final concentrations: kanamycin, 50 μg mL^−1^ for *P*. *ogarae* F113 and insertional mutant strains, and 25 μg mL^−1^ for *E*. *coli* strains, and chloramphenicol, 30 μg mL^−1^, and ampicillin, 100 μg mL^−1^, for the expression plasmids, L-arabinose 0.02% *v*/*v*, isopropyl β-D-thiogalactopyronaside (IPTG) 1 mM, and glucose 0.2% *v*/*v*, according to corresponding vector.

### 2.2. Construction of VgrG5a Auxiliary Module Mutants

Insertional mutants in the *P*. *ogarae* F113; PSF113_0666 (VgrG5a^−^) and _0668 (Tfe8^−^) genes were obtained by introducing by electroporation the plasmid vector pCR2.1^®^-TOPO^®^ (Invitrogen, Waltham, MA, USA) carrying a *ca* 400 bp of the internal region of each of the genes previously detailed. The single recombinant mutants obtained were selected by kanamycin resistance in SA medium and checked by PCR and Southern Blot. For the complementation of the mutations in the PSF113_0666 and PSF113_0668 genes, cosmid pBG200 was used [54]. The cosmid harbors genomic region 790,725 to 809,037 from *P*. *ogarae* F113 [55] from the sequence available in the NCBI databank, which includes the genes PSF113_0666, _0667, _0668, and _0669.

### 2.3. Bioinformatic Analyses

*Pseudomonas* gene sequences for the mutants’ design were obtained from the *Pseudomonas* Genome database [56]. BLASTN of the *vgrG5a* F113 gene was carried out against the nonredundant (nr) NCBI database to search for homologous genes in other members of the *Pseudomonas* genus. Adjacent genes to the hits were recovered to determine the neighborhood of the *vgrG5a* gene in other species. BLASTP analyses were performed on the NCBI website [57] to determine the degree of conservation and the amino acid sequence searches using SMART [58,59]. For the genome analysis of the *Pseudomonas* Genomes Type (Strain), Genome Server (TYGS) [60,61] was employed. Genome Blast Distance Phylogeny (GBDP), implemented in TYGS, was employed to infer genome-to-genome distances. In all cases, the parameters were used at their default values. The neighbor-joining (NJ) phylogenetic tree was constructed using MEGAX [62]. 

PSORTb version 3.0.2 software [63], the Protein Homology/analogy Recognition Engine (Phyre2) server [64], and PredictProtein [65] were used to perform structural-based homology prediction. Phyre2 and AlphaFold Protein Structure Database version 2.3.2 [66] were used to predict the subcellular location of proteins and transmembrane domains, and the prediction of the 3D model and interaction. ChimeraX version 1.6 [67] was employed to structure visualization. 

### 2.4. Interbacterial Competition Assays

Competition assays were performed according to the previously reported protocol [39]. Briefly, kanamycin-resistant derivatives of *P. ogarae* F113 and mutants (predator) and *E. coli* DH5α containing a *lacZ*, kanamycin-resistant pK18*mobsacB* plasmid (prey) were grown overnight in LB medium. Each culture was adjusted to OD_600_ of 1.0, and 100 μL of the predator and 100 μL of the prey strains were mixed and co-cultured for 5 h at 200 rpm and 28 °C. Twenty μL of each culture was spotted onto LB-agar supplemented with 5-bromo-4-chloro-3-indolyl-D-galactopyranoside (X-gal) and kanamycin and incubated at 28 °C. At least three biologically independent experiments were performed. Additionally, serial dilutions of the different assays were plated to quantify the number of CFUs in each of them.

### 2.5. The Toxic and Antitoxic Activity of F113 Tfe8 and Tfi8 Proteins Expressed in E. coli

The *tfe8* and *tfi8*/*tfe8* genes from *P. fluorescens* F113 were amplified by PCR from genomic DNA and cloned into the plasmid vector pT7-7 [68] using the *Nde*I and *Bam*HI restriction sites. Cloning resulted in three plasmids, one of which was empty while the other two carried the toxin (Tfe8) and antitoxin/toxin proteins (Tfi8/Tfe8). These plasmids were transformed into *E. coli* BL21(DE3) expression cells. The induction of expression was performed as previously described [69]. Briefly, cultures were grown from a pre-culture adjusted to the OD_600_ of 1.0 at 28 °C for 4 h, and 20 μL of each culture was spotted in LB plates supplemented with ampicillin and IPTG. The plates were incubated for 24 h at 28 °C. Additionally, the PCR product of the *tfe8* encoding the toxin was cloned into a pNDM220 low-copy number vector [70] named pTfe8, and the *tfi8* gene encoding the antitoxin pair was cloned into a pBAD33 [71] named pTfi8, under the control of the P_BAD_ promoter. Recombinant plasmids were checked by sequencing and then included in *E*. *coli* DH5α by transformation. The *E*. *coli* strain carrying both pTfi8 and pTfe8 plasmids was cultured overnight and then adjusted to an OD_600_ of 0.2. Next, the expression of Tfe8, Tfi8, or both was induced by supplementing the medium with the corresponding regulator: 1 mM IPTG for pNDM220 Tfe8 induction, 0.02% L-arabinose for Tfi8 induction, and 0.2% glucose for Tfi8 repression. Growth was recorded after 24 h by measuring the optical density at OD_600_. The optical density measures were relativized with the measures of *E*. *coli* containing the empty vectors.

### 2.6. Statistical Analyses

The normal distribution of data was checked with the Shapiro–Wilk test, and since the distribution was normal, the data were analyzed with a one-way ANOVA test, where multiple pairwise comparisons between strains were performed with a Tukey HSD test. All data were analyzed with R language version 4.1.1

## 3. Results

### 3.1. An Orphan vgrG5a-tap5a Region Is Present in Strains of the Pseudomonas fluorescens Complex of Species

**Figure 1:**
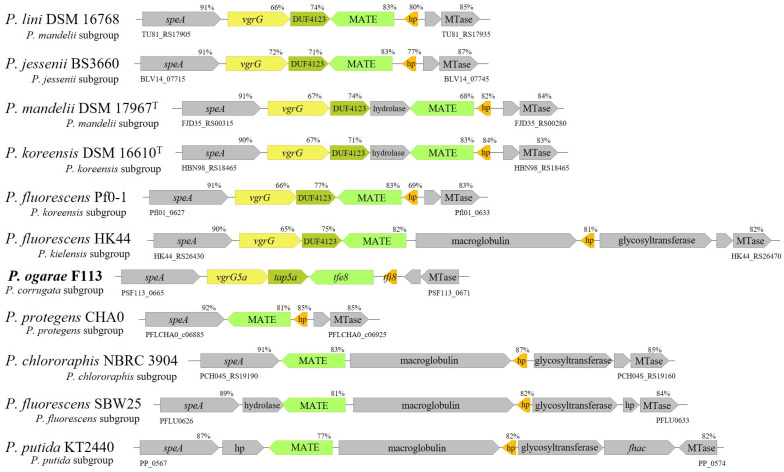
Genetic organization of *vgrG* auxiliary module in *Pseudomonas* genera. The genes are at scale. Values over each gene indicate the sequence identity value in relation with the *vgrG5a* auxiliary module from *P*. *ogarae* F113. hp; hypothetical protein.

The genomic sequence of *P. ogarae* F113 encodes an orphan *vgrG* gene (PSF113_0666) downstream of the *speA* gene (PSF113_0665), an arginine decarboxylase, in the close vicinity of the diguanylate-cyclase encoded by the *gcbA* gene (PSF113_0661). The *vgrG* gene encodes a VgrG5a protein, homologous to T6SS export proteins, and is followed by a gene encoding a DUF4123 protein which is an orthologue of several proteins that act as adaptors for type VI-secreted effectors [21,72]. We have therefore named these genes *vgrG5a* and *tap5a* (PSF113_0667) [44]. The analysis of this region revealed that downstream of these genes and in the opposite transcription direction [73], *P*. *ogarae* F113 encodes one gene which encodes a MATE/Din7 protein domain, similar to the Multidrug and Toxin Compound Extrusion transporter [74] and a small hypothetical protein. Since this genetic organization resembles a T6SS auxiliary module [75], we have named those two genes *tfe8* (PSF113_0668) and *tfi*8 (PSF113_0669) [44], putatively encoding a T6SS effector and its cognate immunity protein. While the orthologues of the putative toxin and immunity genes are widespread within the genus *Pseudomonas,* the gene cluster formed by *vgrG5a* and *tap5a* is present only in a limited number of strains, all belonging to the *Pseudomonas fluorescens* complex of species [45,76]. We have named this region the *speA*-MTase locus, since synteny is maintained by the *speA* gene and the gene encoding a putative MTase protein (Figure 1). Figure 1 shows the genetic organization of the *speA*-MTAse locus in strains belonging to different subgroups of the *P. fluorescens* and *P. putida* groups.

It can be observed that this locus shows high heterogeneity in its organization; downstream *speA*, the *vgrG5a,* and *tap5a* are present in strains of the *Pseudomonas corrugata*, *P*. *mandelii, P*. *jesseni, P*. *koreensis,* and *P*. *kielensis* subgroups. Neither of these genes are present in strains of the *P. fluorescens*, *P*. *protegens*, and *P*. *chlororaphis* subgroups, nor in the *P. putida* group. On the contrary, an orthologue of *tfe8* is present in all the analyzed strains, regardless of the group or subgroup. This gene is always transcribed in the opposite direction of *speA* and is located in different configurations. In most strains harboring the *vgrG5-tap5a* genes, the *tfe8* orthologue is located adjacent to this cluster. In most of the strains that do not contain the *vgrG5a-tap5a*, the *tfe8* orthologue is adjacent to *speA*. However, in a few strains, another gene, putatively encoding a hydrolase, is located adjacent to the *tfe8* orthologue. Regarding *tfi8*, its orthologues are located just upstream *tfe8* and in the same sense of the transcription in most of the strains that also contain *vgr5a* and *tap5a*. In most of the other strains, both genes are separated by another gene, encoding a putative macroglobulin. These data show that the *speA*-MTase locus has undergone several restructurations during evolution by acquiring new genes and eliminating others. The acquisition of the *vgrG5a-tapA5a* pair seems to be a relevant evolutionary landmark indicating a gain of function.

**Figure 2:**
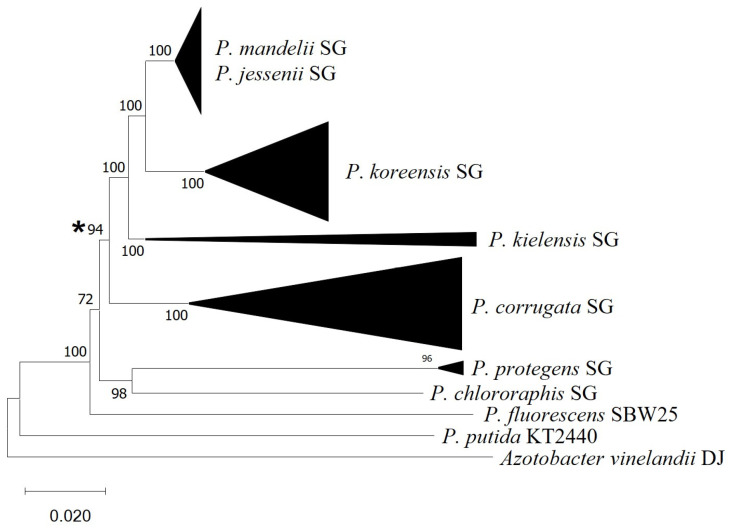
GBDP-based phylogeny of fifty genomes belonging to *Pseudomonas* groups. Subtree composed by major subgroups has been compressed and their names indicated. Asterisk indicates the genetic acquisition event of *vgrG* and *tap* genes. MEGAX was employed for the visualization and edition of the tree. Genomes employed are listed in Table S2.

In order to investigate the prevalence of this gain of function, we have investigated the presence of the *vgrG5a-tap5a* genes in a larger number of strains belonging to the *P. corrugata*, *P*. *mandelii*, *P*. *jesseni*, *P*. *koreensis,* and *P*. *kielensis* subgroups. The results have shown that the *vgrG5a-tap5a* are present in the vast majority of strains from these groups with genomic sequences available (Supplementary Table S2). We have not found these genes in any strains outside these subgroups. A phylogenomic tree containing all the strains tested (Figure 2 and Figure S1) shows that the presence of the *vgr5a-tap5a* occurs in a monophyletic group which includes the *P. fluorescens* subgroups *P*. *corrugata, P*. *mandelii, P*. *jesseni, P*. *koreensis,* and *P*. *kielensis*.

These results indicate that the acquisition of *vgrG5a-tap5a* has occurred once in evolution, in a common ancestor to all these subgroups. Other *P. fluorescens* subgroups, such as *P*. *fluorescens*, *P*. *protegens,* and *P*. *chlororaphis*, that do not contain the genes, are paraphyletic with the other subgroups (Figure 2 and Figure S1). Other restructurations within this locus are more difficult to date, but the physical separation between *tfe8* and *tfi8* seems to be an ancestral trait, and the loss of the intertwining gene has likely evolved with the acquisition of the *vgrG5-tap5a* genes. As a result of these two processes, a genetic region that appears to have genes encoding a VgrG protein, a Type VI adaptor protein, and a putative secreted effector and its cognate immunity protein has evolved in a monophyletic group of pseudomonads. Conversely to the other Tfes encoded by the *P*. *ogarae* F113 genome [44], Tfe8 does not resemble any known type VI effector or harbor any previously described T6SS-related domains, such as the PAAR or MIX domains [77].

### 3.2. The VgrG5a-Tap5a Cluster Is Involved in Bacterial Killing

**Figure 3:**
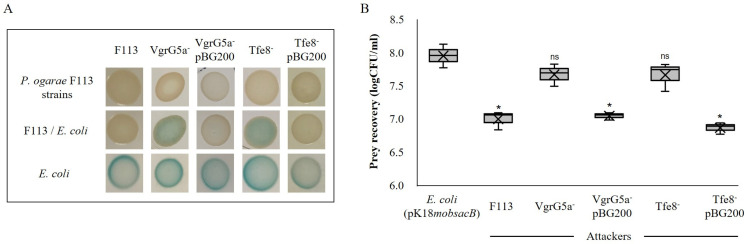
Bacterial killing profile of the *vgrG5a* auxiliary module between *P. ogarae* F113 mutants and *E. coli* strain carrying pK18*mobsacB* expressing *lacZ*. (**A**) Bacterial patches obtained from the bacteria–bacteria interaction, where the blue color on LB plates (X-gal and kanamycin) indicates *E. coli* growth. The top row shows the growth of *P. ogarae* control or its insertional mutant strains. The middle row shows the growth of mixed *P. ogarae*/*E. coli* cultures after 5 h of co-incubation. Bottom row shows the *E. coli* reference culture. (**B**) Bacterial killing quantification. Box and whiskers plots indicating the amount of prey recovery from each attacker expressed as logCFU/mL. Significance was calculated using ANOVA test (* *p* value < 0.01); ns, not significant differences when compared to non-competing *E*. *coli*. X symbol in each box indicates the mean in each sample.

Considering the resemblance of the *P*. *ogarae* F113 *speA*-MTase locus with a T6SS effector delivery system, we decided to test the relation of this locus with bacterial killing. For that, we generated insertion mutants affecting either the *vgrG5a* gene or the *tfe8* gene, putatively encoding the type VI effector. In order to test killing, we used two approaches [5]. First, we used a non-quantitative patch method (Figure 3A) using a *lacZ* tagged *E. coli* strain as the prey and *P*. *ogarae* F113 insertional mutants as the predators. Therefore, *E. coli* killing was observed as a lack of blue color generated by *E. coli* pK18*mobsacB* in the presence of X-gal in LB plates. Figure 3A shows the killing ability for the F113 wild-type and an impairment in the killing for both mutants.

The complementation of the mutants with a cosmid (pBG200) from a F113 library, previously isolated, which spans from upstream of the *gcbA* gene and contains the *speA*-Mtase locus, fully restored the killing capacity of the mutants. In the second quantitative approach (Figure 3B), we used the same prey and predator strains, but the killing activity was quantified by the recovery of the prey cells after contact with the predator strain.

The results obtained were similar to that of the previous experiment, since prey recovery was ten times lower after contact with the wild-type strain than that after contact with either of the mutant strains. The mutants recovered their killing ability after complementation with pBG200 to the same level than the wild-type strain.

### 3.3. Tfe8 and Tfi8 Are a T6SS Effector and Immunity Protein

**Figure 4:**
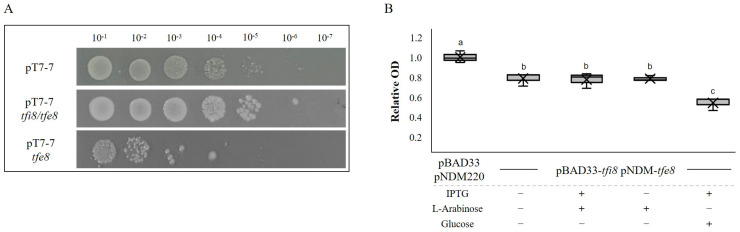
Tfe8/Tfi8 constitute an effector–immunity pair. (**A**) Serial dilution of bacterial survival after the expression of cloned *tfe8* and *tfi8/tfe8* in *E. coli* BL21(DE3) cells. The top row corresponds to the control strain carrying an empty pT7-7 vector, the middle row corresponds to the induced cells of the cloned *tfi8/tfe8* genes in pT7-7, and the lower row to the induced cells of the cloned *tfe8* gene. All the strains present similar survival without induction. (**B**) Growth inhibition assays. Box and whiskers plot showing the growth of the *E*. *coli* DH5α-*tfi8*/*tfe8* strain containing pBAD33-*tfi8* and pNDM220-*tfe8* plasmids in liquid culture. Significance was calculated using ANOVA with a Tukey test, where a and b (*p* value < 0.01), b and c (*p* value < 0.001), and between a and c (*p* value < 0.001). Optical density was recorded for over 24 h. Optical density was relativized to the of *E*. *coli* DH5α carrying the empty plasmids pBAD33 and pNDM220.

Since both the VgrG5a and Tfe8 proteins are implicated in bacterial killing, we tested the role of Tfe8 and Tfi8 by ectopic expression in *E. coli* cells. We also used two approaches for this test. In the first approach, we cloned either the *tfe8* gene or the *tfi8-tfe8* genes in the pT7-7 plasmid. Serial dilutions of *E. coli* cells harboring either the empty vector or the vector containing each of the two constructions were plated on a nutrient medium. Figure 4A shows that cells expressing the *tfe8* gene grew two orders of magnitude less than cells with the empty vector. Furthermore, construction containing *tfi8* and *tfe8* grew to a level similar to that of cells containing the empty vector, indicating that cells express both *tfi8* and *tfe8.*

In the second approach, we cloned *tfe8* in plasmid pNDM220 under the control of an IPTG inducible promoter and *tfi8* in pBAD33 under the control of the *araB* promoter, that is inducible by arabinose and repressed by glucose. Both constructs were introduced in DH5α *E. coli* and a control strain was generated by introducing both empty vectors. As observed in Figure 4, the growth of the *E. coli* strain harboring both constructs was reduced in respect to the control strain under all tested conditions. These results indicate that even in the absence of induction, Tfe8 is produced, resulting in toxicity for the cells. The *araB* promoter seems also to be leaky in the absence of induction. However, when the *araB* promoter is repressed by glucose, the production of the putative immunity protein appears to be reduced, since upon induction with IPTG, the toxicity is higher, meaning that only the effector and not the immunity protein are produced.

Taken together, these results strongly suggest that *tfe8* encodes a T6SS effector, probably secreted as a cargo by VgrG5a with the help of the adaptor protein Tap5a, and that Tfi8 is its putative cognate immunity protein.

**Figure 5:**
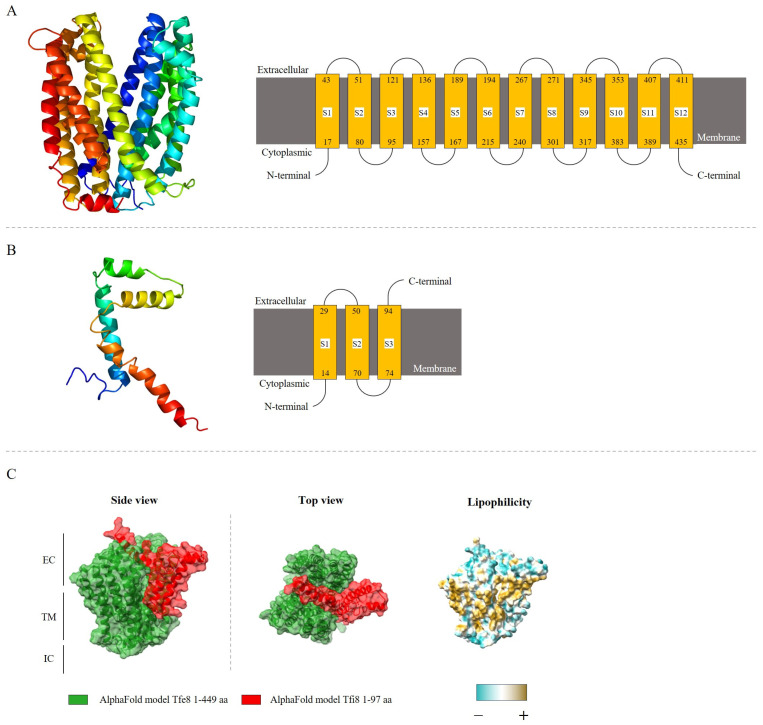
Transmembrane helix prediction and topology adopted for the proteins analyzed through Phyre2 web portal for protein modeling. (**A**) PSF113_0668 (Tfe8). (**B**) PSF113_0669 (Tfi8). (**C**) Tfe8-Tfi8 predicted protein interaction by AlphaFold-Multimer. The complex has transmembrane, extracellular, and intracellular domains. Lateral and top view are present. Lipophilicity is indicated in the figure by colors, from more hydrophilic (blue) to more lipophilic (golden) surface. Protein structure was visualized with ChimeraX. EC; ExtraCellular, TM; TransMembrane, IC; ItraCellular. Figure S2 includes a QR code that allows the view of the interaction model in motion on the X and Y axis.

The analysis of the subcellular localization of the putative immunity protein pair, Tfi8 and Tfe8, was made through a bioinformatics approach using the prediction tools PSORTb version 3.0.2, Phyre2 version 2.0, and AlphaFold version 2.3.2. These analyses indicate that both proteins are transmembrane proteins (Figure 5), where the toxin protein Tfe8 possesses between ten and twelve transmembrane domains, depending on the bioinformatics approach employed. The Tfe8 protein could form a possible pore in the target bacterial cell membrane (Figure 5A), and the analysis of the protein residues from Tfe8 revealed that 98% of this sequence has been modeled with 100% confidence by the single highest scoring template, and according to the Research Collaboratory for Structural Bioinformatics RCSB protein data bank, these template structures are related with the Multidrug and Toxin Compound Extrusion (MATE) transporter present in *V. cholerae* [78] and *E. coli* [79].

The predicted structure for the Tfi8 immunity protein presents three transmembrane domains, with an elongated tertiary structure (Figure 5B), and 61 residues of the putative immunity protein Tfi8 (63% of sequence) have been modeled with 38.1% confidence by the single highest scoring template. Model predictions employing AlphaFold predict the binding of one putative immunity Tfi8 to the pore generated by the Tfe8 protein. No signal peptides are found in any of these proteins, suggesting that they are not translocated to the cytoplasmic membrane through a general secretion pathway, where the analysis of these proteins also corroborates the idea of the transmembrane location of both proteins.

## 4. Discussion

Besides complete T6SSs, many bacteria encode accessory modules characterized by the presence of genes encoding T6SS proteins such as Hcp or VgrG, plus genes encoding other proteins including effectors and immunity proteins [75,80]. In the case of *P. ogarae* F113, its genome encodes three complete T6SSs and five accessory modules, each containing a gene encoding a different VgrG protein. We have previously shown that at least two of the T6SSs, F1-T6SS and F3-T6SS, are functional for bacterial killing [44]. Here, we analyze the role of one of the accessory modules regarding bacterial killing. The analyzed module contains a gene encoding a VgrG5a protein followed by a gene encoding a DUF4123 protein. DUF4123 proteins have been described as adaptor proteins for the secretion of cargo effectors by VgrG proteins [21]. Following this gene and in the opposite transcription direction, there are two genes encoding a MATE/DinF protein (*tfe8*) and a small hypothetical protein (*tfi8*). PSORTb predicts that both proteins are transmembrane proteins with transmembrane helices. No signal peptides are found in any of these proteins, suggesting that they are not translocated to the cytoplasmic membrane through a general secretion pathway. Phyre2 analysis of these proteins (Figure 5) also indicates the transmembrane location of both proteins. Furthermore, AlphaFold predicts an interaction of these two proteins in which Tfi8 could block the Tfe8 pore in the extracellular side, explaining its putative role as an immunity protein.

The four proteins in the auxiliary module present the structure of a typical T6SS auxiliary module, with a VgrG protein, an adaptor protein, and a putative effector and its cognate immunity protein. Similar gene arrangements have been found in other bacteria [21,35]. MATE proteins constitute a superfamily of multidrug and toxic compounds extrusion [81,82] that is divided into DinF, NorM, and eukaryotic families [83,84]. MATE/DinF-encoding genes are widespread among pseudomonads and, in many cases, are not genetically linked with genes related with secretion systems. However, a MATE-encoding gene has been found to be under the control of the Type Three Secretion system regulator HrpL in *P. syringae* pv. tomato DC3000, and its deletion resulted in reduced virulence [85]. This result suggests that MATE proteins can act as effectors if secreted through a specialized system.

We have shown here that this accessory module is only present within the *P. fluorescens* group and that it is conserved in a monophyletic branch that includes the *P*. *corrugata*, *P*. *koreensis, P*. *mandelii, P*. *jesseni,* and *P*. *kielensis* subgroups. These results suggest that the acquisition of the VgrG-Tap module, and therefore the possibility to secrete the MATE-DinF protein (Tfe8), has occurred only once in evolution, in a common ancestor of the subgroups carrying this module. The lack of close homologues of the *vgrG5a* and *tap5a* genes within the pseudomonads may indicate that its origin is from outside the genus. It is interesting to note that a limited number of strains from the *P. syringae* group, with genomes deposited in the Pseudomonas Genome Database [56], harbor a different VgrG-encoding gene in this locus, suggesting that these strains might also secrete a MATE/DinF protein.

We have also shown the importance of this accessory module in bacterial killing, since the mutation of either the VgrG- or the Tfe8-encoding genes resulted in a significant reduction of the *E. coli* killing ability in two different bacterial killing assays. In this strain, we previously showed that mutations affecting either of the two T6SSs (F1 and F3) also showed a reduction in killing [44], indicating that the auxiliary module also plays a relevant role in bacterial killing. The results presented here are also the first to show a role in the bacterial killing of a T6SS effector in this strain. Within pseudomonads, the role of auxiliary modules in bacterial killing has been previously shown in *P. protegens*, where two of these modules, one of them distantly located from the core T6SS genes, were shown to affect the *Enterobacter* population in an insect larvae gut, resulting finally in larvae death [46]. We have also shown the role of the Tfe8 protein as a putative T6SS effector by the ectopic expression of this gene in *E. coli* (Figure 4). The expression of *tfe8* from two different vectors resulted in the reduced growth of *E. coli* cells, indicating Tfe8 toxicity. To our knowledge, it is the first MATE/DinF protein to be shown as a putative T6SS effector. Regarding its cognate immunity protein, the results presented here have shown that Tfi8 can be identified as a putative immunity protein, since repressing *tfi8* expression while expressing *tfe8* resulted in a higher impact on *E. coli* growth (Figure 4B).

## 5. Conclusions

The results presented here show that the *speA*-mTase locus in *P. ogarae* F113 harbors a T6SS accessory module formed by VgrG5a, TapA5a, Tfe8, and Tfi8. They also show that only a monophyletic group of pseudomonads harbor this accessory module that provides the ability to kill *E. coli* cells upon contact. A MATE/DinF protein has been shown to act as a putative type six effector that is probably secreted through T6SSs and acting as a pore-forming toxin.

## Data Availability

The data supporting the findings of this study are available within the article and its Supplemental Material. Raw data that support the findings of this study are available from the corresponding author upon reasonable request.

## Supplementary Materials

The following supporting information can be downloaded at: https://www.mdpi.com/article/10.3390/genes14111979/s1, Table S1: The bacterial strains and plasmids employed in this study; Table S2: Genomes employed in the GBDP phylogeny tree of *Pseudomonas* groups Figure 1 and Figure S1; Figure S1: GBDP-based phylogeny of 186 genomes belonging to *Pseudomonas* groups; Figure S2: QR code of Tfi8/Tfe8 interaction model.
