# Supplementary material for: An Orphan VrgG Auxiliary Module Related to the Type VI Secretion Systems from Pseudomonas ogarae F113 Mediates Bacterial Killing
Source: Genes (Basel). 2023 Oct 24;14(11):1979. doi: 10.3390/genes14111979 (PMC10671463; doi:10.3390/genes14111979)
Supplement: Supplementary file 1 [file genes-14-01979-s001.zip › genes-2663166-supplementary.pdf]

Supplementary Information

An orphan VrgG auxiliary module related to the type VI Secretion Systems from *Pseudomonas ogarae* F113 mediates bacterial killing.

David Durán[1], David Vazquez-Arias[1], Esther Blanco-Romero[1], Daniel Garrido-Sanz[1,2], Miguel Redondo-Nieto[1], Rafael Rivilla[1] and Marta Martín[1*]

[1] Departamento de Biología, Facultad de Ciencias, Universidad Autónoma de Madrid, Darwin, 2, 28049 Madrid, Spain.
[2] Department of Fundamental Microbiology, University of Lausanne, 1015 Lausanne, Switzerland.

Content

Supplementary Tables 1 and 2
Supplementary Figures 1 and 2

Table S1. The bacterial strains and plasmids employed in this study

Strains	Genotype	Source or Reference
P. fluorescens		
F113	wild type strain, Rif^R	[89]
VgrG5a$^-$	PSF113_0666:: pCR2.1-TOPO, Rif^R Km^R	This work
VgrG5a$^-$ (pBG200)	PSF113_0666:: pCR2.1-TOPO, Rif^R Km^R Tc^R complemented with the cosmid pBG200	This work
Tfe8$^-$	PSF113_0668:: pCR2.1-TOPO, Rif^R Km^R	This work
Tfe8$^-$ (pBG200)	PSF113_0668:: pCR2.1-TOPO, Rif^R Km^R Tc^R complemented with the cosmid pBG200	This work
E. coli		
DH5α	*recA1 endA1 gyrA96 thi hsdRl7 supE44 relA1* Δ(*lacZYA-argF*)*U169* (Φ80*lacZ*ΔM15) *deoR phoA*	[87] Gibco-BRL
BL21(DE3)	*fhuA2 [lon] ompT gal (λ DE3) [dcm]* Δ*hsdS* *λ DE3 = λ sBamHIo* Δ*EcoRI-B* *int::(lacI::PlacUV5::T7 gene1) i21* Δ*nin5*	New England BioLabs inc.
pT7-7 *tfe8*	PSF113_0668 cloning in pT7-7 expressed in BL21(DE3) competent cells, Amp^r	This work
pT7-7 *tfi8*/*tfe8*	PSF113_0668-0669 cloning in pT7-7 expressed in BL21(DE3) competent cells, Amp^r	This work
pBAD33 - pNDM220	Empty PNDM220 and PBAD33 expressed in DH5α competent cells, Amp^r Cm^r	This work
pBAD33-*tfi8* pNDM220-*tfe8*	PSF113_0669 cloning in pBAD33 and PSF113_0668 cloning in pNDM220 and expressed in DH5α competent cells, Amp^r Cm^r	This work
Plasmids		
pT7-7	General cloning vector, Escherichia coli K12 DH1 derivative; pT7phi10 promoter, Amp^r	[86]
pNDM220	Low-copy-number cloning vector, pA1/O4/O3 promoter, IPTG- inducible. Amp^r	[70]
pBAD33	High-copy-number cloning vector, P_{BAD} promoter, L-arabinose – inducible, glucose repressible. Cm^r	[71]
pCR2.1-TOPO	PCR product cloning vector; Ap^r, Km^r	Invitrogen
pK18*mobsacB*	Integrative vector pUC18 derivative; *lacZ mob* site *sacB*, Km^r	[88]
Cosmid		
pBG200	Cosmid harbours genomic region 790725 to 809037 from *P.* ogarae F113	This work

Table S2. Genomes employed in the GBDP phylogeny tree of *Pseudomonas* groups Figure 1 and Figure S1.

Strain	Refseq assembly accession
Azotobacter vinelandii DJ *	GCF_000021045.1
Pseudomonas lini DSM 16768 *	GCF_001042905.1
Pseudomonas brassicacearum 51MFCVI2.1 *	GCF_000510785.1
Pseudomonas brassicacearum DF41 *	CP007410.1
Pseudomonas brassicacearum NFM421 *	GCF_000194805.1
Pseudomonas brassicacearum PA1G7 *	GCF_000800585.1
Pseudomonas brassicacearum PP1 210F *	GCF_000785375.1
Pseudomonas chlororaphis LMG 5004 *	GCF_002091535.1
Pseudomonas fluorescens AU11114 *	GCF_001020875.1
Pseudomonas fluorescens AU5633 *	GCF_001020865.1
Pseudomonas fluorescens C1 *	GCF_000967945.1
Pseudomonas fluorescens C2 *	GCF_000802965.1
Pseudomonas fluorescens C3 *	GCF_000967955.1
Pseudomonas fluorescens HK44 *	GCF_000217955.2
Pseudomonas fluorescens MEP34 *	GCF_000834545.1
Pseudomonas fluorescens PA3G8 *	GCF_000800625.1
Pseudomonas fluorescens Pf0-1 *	GCF_000012445.1
Pseudomonas fluorescens Q2-87 *	GCF_000281895.1
Pseudomonas fluorescens Q8r1-96 *	GCF_000263695.1
Pseudomonas fluorescens R124 *	GCF_000292795.1
Pseudomonas fluorescens SBW25 *	GCF_000009225.1
Pseudomonas fluorescens SF39a *	GCF_000817895.1
Pseudomonas fluorescens SF4c *	GCF_000817905.1
Pseudomonas frederiksbergensis SI8 *	GCF_000802155.2
Pseudomonas kilonensis 1855-344 *	GCF_000968575.1
Pseudomonas mandelii 36MFCvi1.1 *	GCF_000381285.1
Pseudomonas mandelii JR-1 *	GCF_000257545.3
Pseudomonas mediterranea CFBP 5447 *	GCF_000774145.1
Pseudomonas moraviensis R28-S *	GCF_000512275.1

***** strains included in Figure 1

Strain	Refseq assembly accession
Pseudomonas ogarae F113 *	CP003150.1
Pseudomonas protegens CHA0 *	GCF_000397205.1
Pseudomonas protegens pf5 *	GCF_008807675.1
Pseudomonas putida KT2440 *	GCF_000007565.2
Pseudomonas sp. 11/12A *	GCF_000800055.1
Pseudomonas sp. 35MFCvi1.1 *	GCF_000378525.1
Pseudomonas sp. 45MFCol3.1 *	GCF_000382025.1
Pseudomonas sp. GM25 *	GCF_000282255.1
Pseudomonas sp. GM30 *	GCF_000282275.2
Pseudomonas sp. GM41 *	GCF_000282315.2
Pseudomonas sp. H1h *	GCF_000633255.1
Pseudomonas sp. PTA1 *	GCF_000745605.1
Pseudomonas sp. RIT288 *	GCF_000631985.1
Pseudomonas sp. UFB2 *	CP011020.1
Pseudomonas sp. URHB0015 *	GCF_000620245.1
Pseudomonas sp. URIL14HWK12:I6 *	GCF_000514195.1
Pseudomonas sp. URIL14HWK12:I7 *	GCF_000514275.1
Pseudomonas sp. URMO17WK12:I12 *	GCF_000514395.1
Pseudomonas sp. UW4 *	GCF_000316175.1
Pseudomonas umsongensis 20MFCvi1.1 *	GCF_000377725.1
Pseudomonas umsongensis UNC430CL58Col *	GCF_000620285.1
Pseudomonas ekonensis COR58	GCF_019145435.1
Pseudomonas kielensis MBT-1	GCF_014236655.1
Pseudomonas aestus CMAA1215	GCF_000474765.1
Pseudomonas allii MAFF 301514	GCF_013392005.1
Pseudomonas allokribbensis LMG31525	GCA_014863605.1
Pseudomonas alvandae SWRI17	GCA_019141525.1
Pseudomonas antarctica LMG 22709	GCF_900103795.1
Pseudomonas anuradhapurensis RD8MR3	GCF_014269225.2

Table S2. (continuation)

Strain	Refseq assembly accession
Pseudomonas aromaticivorans MAP12	GCA_019097855.1
Pseudomonas arsenicoxydans CECT 7543	GCF_900103875.1
Pseudomonas asgharzadehiana SWRI132	GCF_019139815.1
Pseudomonas asiatica JCM 32716	GCA_009932335.1
Pseudomonas atacamensis M7D1	GCF_004801935.1
Pseudomonas atagonensis PS14	GCF_011369485.1
Pseudomonas avellanae BPIC 631	GCF_000444135.1
Pseudomonas avellanae JCM 11937	GCF_014646595.1
Pseudomonas aylmerensis S1E40	GCF_003031665.1
Pseudomonas azerbaijanoccidentalis SWRI74	GCF_019145495.1
Pseudomonas azerbaijanorientalis SWRI123	GCF_019139795.1
Pseudomonas azotoformans LMG 21611	GCF_900103345.1
Pseudomonas baetica LMG 25716	GCF_002813455.1
Pseudomonas bananamidigenes BW11P2	GCF_001679645.1
Pseudomonas bijieensis L22-9	GCF_013347965.1
Pseudomonas boanensis CCUG 62977	GCF_018704125.1
Pseudomonas botevensis COW3	GCF_019145475.1
Pseudomonas brassicacearum LMG 21623	GCF_900103245.1
Pseudomonas canavaninivorans HB002	GCF_016405165.1
Pseudomonas cannabina ICMP 2823	GCF_001400175.1
Pseudomonas carbonaria CIP 111764	GCF_904061905.1
Pseudomonas caricapapayae ICMP 2855	GCA_001400735.1
Pseudomonas caspiana FBF102	GCF_002158995.1
Pseudomonas cerasi 58	GCF_900074915.1
Pseudomonas chlororaphis subsp. *aurantiaca* DSM 19603	GCF_003851835.1
Pseudomonas chlororaphis subsp. *aureofaciens* NBRC 3521	GCF_000813225.1
Pseudomonas chlororaphis subsp. *piscium* DSM 21509	GCF_001269555.1
Pseudomonas congelans DSM 14939	GCF_900103225.1
Pseudomonas corrugata DSM 7228	GCA_001269905.1
Pseudomonas crudilactis UCMA 17988	GCF_013433315.1
Pseudomonas cyclaminis MAFF 301449	GCF_015163715.1
Pseudomonas lalucatii R1b-54	GCA_018398425.1

Strain	Refseq assembly accession
Pseudomonas entomophila L48	GCF_000026105.1
Pseudomonas extremaustralis 14-3	GCF_000242115.1
Pseudomonas extremorientalis LMG 19695	GCF_001870465.1
Pseudomonas farris SWRI79	GCF_019145235.1
Pseudomonas fildesensis KG01	GCF_001050345.1
Pseudomonas fitomaticsae FIT81	GCF_021018765.1
Pseudomonas folii DOAB 1069	GCA_014357575.1
Pseudomonas frederiksbergensis LMG 19851	GCF_900105495.1
Pseudomonas furukawaii KF707	GCF_000262065.1
Pseudomonas germanica FIT28	GCF_019614655.1
Pseudomonas glycinae MS586	GCF_001594225.1
Pseudomonas gozinkensis LMG 31526	GCA_014863585.1
Pseudomonas granadensis LMG 27940	GCF_900105485.1
Pseudomonas gregormendelii LMG 28632	GCF_017114825.1
Pseudomonas grimontii DSM 17515	GCF_007858185.1
Pseudomonas guangdongensis CCTCCAB2012022	GCF_900105885.1
Pseudomonas hamedanensis SWRI65	GCF_014268595.2
Pseudomonas indica NBRC 103045	GCF_002091635.1
Pseudomonas indoloxydans JCM 14246	GCF_003052605.1
Pseudomonas inefficax JV551A3	GCF_900277125.1
Pseudomonas iranensis SWRI54	GCF_014268585.2
Pseudomonas iridis P42	GCA_017973755.1
Pseudomonas izuensis IzPS43 3003	GCF_009861505.1
Pseudomonas jessenii DSM 17150	GCF_002236115.1
Pseudomonas kermanshahensis SWRI100	GCF_014269205.1
Pseudomonas khorasanensis SWRI153	GCF_014268505.1
Pseudomonas kilonensis DSM 13647	GCF_001269885.1
Pseudomonas kitaguniensis MAFF 212408	GCF_009296165.1
Pseudomonas koreensis JCM 14769	GCF_014646955.1
Pseudomonas kribbensis KCTC 32541	GCF_003352185.1
Pseudomonas kurunegalensis RW1P2	GCF_014269245.1
Pseudomonas putida NBRC 14164	GCF_000412675.1

Table S2. (continuation)

Strain	Refseq assembly accession
Pseudomonas laurylsulfatiphila AP3 16	GCF_002934665.1
Pseudomonas laurylsulfativorans AP3 22	GCF_002906155.1
Pseudomonas libanensis DSM 17149	GCF_001439685.1
Pseudomonas linyingensis LMG 25967	GCF_900109175.1
Pseudomonas lurida LMG 21995	GCF_002563895.1
Pseudomonas mandelii LMG 21607	GCF_900106065.1
Pseudomonas mangiferae DMKU BBB3-04	GCF_007109405.1
Pseudomonas marginalis DSM 13124	GCF_007858155.1
Pseudomonas marginalis ICMP 3553	GCF_024169225.1
Pseudomonas marvdashtae SWRI102	GCA_014268655.1
Pseudomonas meliae CFBP 3225	GCF_000935675.1
Pseudomonas migulae NBRC 103157	GCF_002091715.1
Pseudomonas mohnii DSM 18327	GCF_900105115.1
Pseudomonas monsensis PGSB 8459	GCF_014268495.2
Pseudomonas monteilii DSM 14164	GCF_000621245.1
Pseudomonas moorei DSM 12647	GCF_900102045.1
Pseudomonas moraviensis LMG 24280	GCF_900105805.1
Pseudomonas morbosilactucae MAFF 302030	GCA_023241715.1
Pseudomonas mucidolens LMG 2223	GCF_900106045.1
Pseudomonas mucoides P154a	GCF_015461845.1
Pseudomonas muyukensis COW39	GCF_019139535.1
Pseudomonas neuropathica P155	GCF_015461835.1
Pseudomonas oryzae KCTC 32247	GCF_900104805.1
Pseudomonas oryzicola RD9SR1	GCF_014269185.1
Pseudomonas pergaminensis 1008	GCF_024112395.1
Pseudomonas petroselini MAFF 311094	GCF_021166635.1
Pseudomonas piscicola P50	GCA_015351605.1
Pseudomonas piscis KCTC 72033	GCA_009380155.1
Pseudomonas plecoglossicida DSM 15088	GCF_000688275.1
Pseudomonas prosekii LMG 26867	GCF_900105155.1
Pseudomonas proteolytica LMG 22710	GCF_900105955.1
Azotobacter chroococcum DSM 2286	GCA_004339665.1

Strain	Refseq assembly accession
Pseudomonas reinekei MT1	GCF_001945365.1
Pseudomonas rhizophila S211	GCA_003033885.1
Pseudomonas rustica DSM 112348	GCF_018336155.1
Pseudomonas sagittaria JCM 18195	GCF_900115715.1
Pseudomonas saponiphila DSM 9751	GCF_900105185.1
Pseudomonas savastanoi ICMP 4352	GCF_001401285.1
Pseudomonas sessilinigenes CMR12a	GCF_019139855.1
Pseudomonas shahriarae SWRI52	GCF_014268455.1
Pseudomonas silesiensis A3	GCA_001661075.1
Pseudomonas siliginis SWRI31	GCF_019145195.1
Pseudomonas simiae CCUG 50988	GCF_900111895.1
Pseudomonas syringae KCTC 12500	GCF_000507185.1
Pseudomonas taiwanensis DSM 21245	GCF_000425785.1
Pseudomonas tehranensis SWRI196	GCA_014268615.1
Pseudomonas tensinigenes ZA 5.3	GCF_014268445.2
Pseudomonas thermotolerans DSM 14292	GCF_000364625.1
Pseudomonas thivervalensis LMG 21626	GCA_001637285.1
Pseudomonas tritici SWRI145	GCF_014268275.3
Pseudomonas triticicola SWRI88	GCF_019145375.1
Pseudomonas umsongensis DSM 16611	GCF_002236105.1
Pseudomonas uvaldensis 20TX0172	GCA_021271205.1
Pseudomonas vancouverensis LMG 20222	GCF_900105825.1
Pseudomonas veronii DSM 11331	GCF_001439695.1
Pseudomonas viciae 11K1	GCA_004786035.1
Pseudomonas vlassakiae RW4S2	GCF_014269035.2
Pseudomonas xantholysinigenes RW9S1A	GCF_014268885.2
Pseudomonas yamanorum LMG 27247	GCF_900105735.1
Pseudomonas zanjanensis SWRI12	GCA_014268745.1
Pseudomonas zarinae SWRI108	GCF_014268695.2
Pseudomonas zeae OE 48.2	GCF_014268485.2
Azotobacter beijerinckii DSM 378	GCA_900110885.1
Azotobacter chroococcum subsp. *isscasi* P205	GCA_004327895.1

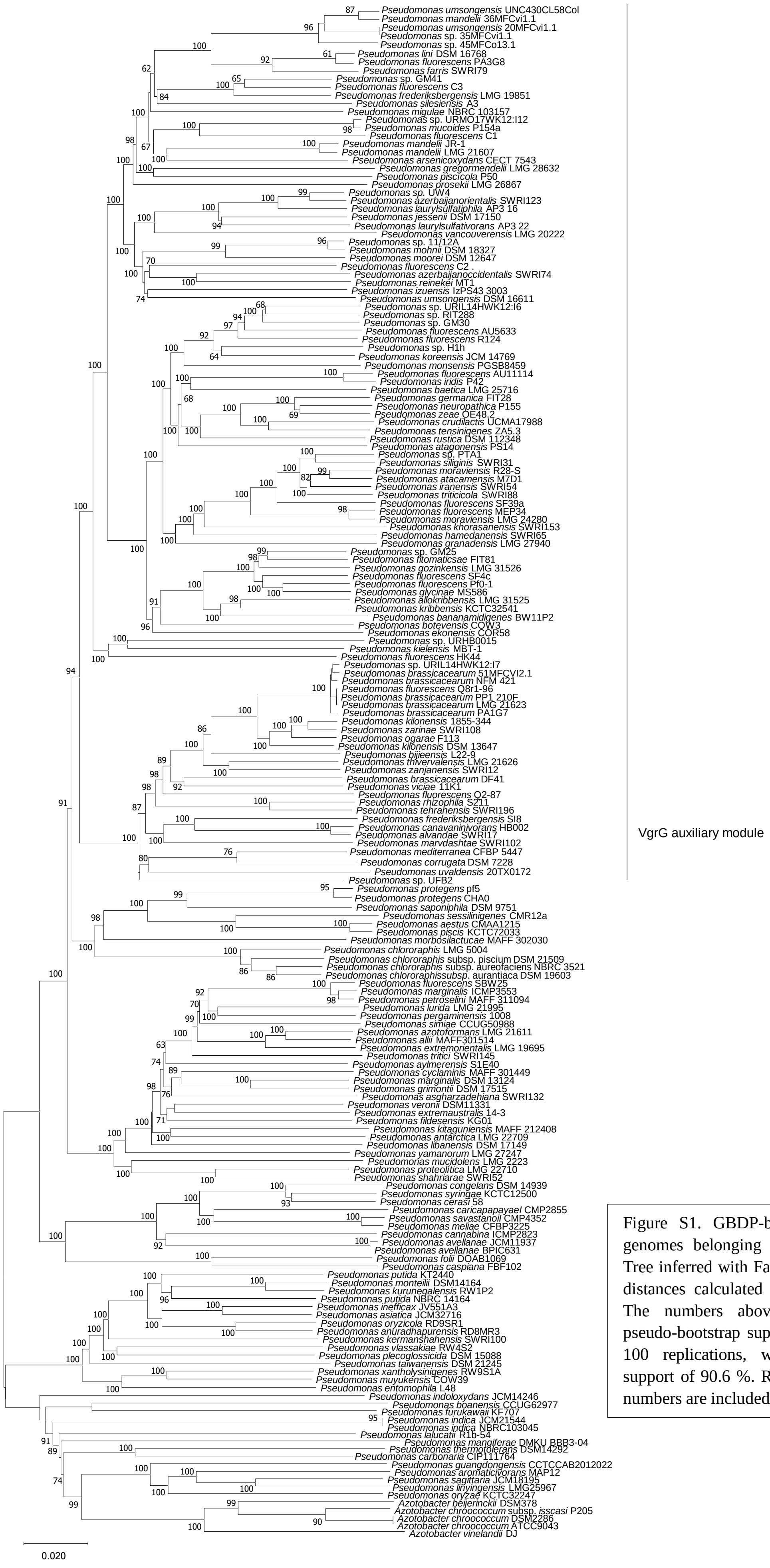

Figure S1. GBDP-based phylogeny of 186 genomes belonging to *Pseudomonas* groups. Tree inferred with FastME 2.1.6.1 from GBDP distances calculated from genome sequences. The numbers above branches are GBDP pseudo-bootstrap support values > 60 % from 100 replications, with an average branch support of 90.6 %. Refseq assembly accession numbers are included in Table S1.

Figure S2. QR code of Tfi8/Tfe8 interaction model
